# Hypoxia and hyperoxia potentiate PAF receptor‐mediated effects in newborn ovine pulmonary arterial smooth muscle cells: significance in oxygen therapy of PPHN

**DOI:** 10.14814/phy2.12840

**Published:** 2016-06-28

**Authors:** Mona Hanouni, Gilberto Bernal, Shaemion McBride, Vincent Reginald F. Narvaez, Basil O. Ibe

**Affiliations:** ^1^ Division of NeonatologyDepartment of PediatricsLos Angeles Biomedical Research Institute at Harbor‐UCLA Medical CenterTorranceCalifornia

**Keywords:** cAMP, hyperoxia, PAF receptor, PKA, prostacyclin

## Abstract

Platelet‐activating factor (PAF) acting via its receptor (PAFR) is implicated in the pathogenesis of persistent pulmonary hypertension of the newborn (PPHN). Effects of long‐term oxygen therapy on newborn lung are not well understood; therefore, we studied the effect of oxygen tension on ovine newborn pulmonary artery smooth muscle cells (NBPASMC). Our global hypothesis is that PPHN results from failure of newborn lamb pulmonary system to downregulate PAFR activity or to upregulate vasodilatory cyclic nucleotides (Cnucs) activity. NBPASMC from newborns 6–12 days old were studied in vitro at three different oxygen tensions (pO_2_, [Torr]: hypoxia, <40; normoxia, 80–100; and hyperoxia, >100 Torr often clinically imposed upon newborns with PPHN). PAFR‐ and Cnucs mediated effects were determined. *PAFR* and *PKA Cα* mRNA expression as well as prostacyclin, thromboxane, cAMP production, and DNA synthesis was studied to assess PAFR‐mediated hypertrophy and/or hyperplasia. Hypoxia and hyperoxia increased specific PAFR binding. PAF treatment during hyperoxia increased *PAFR* gene, but decreased *PKA‐Cα* gene expression. Hypoxia and hyperoxia increased NBPASMC proliferation via PAFR signaling. Baseline prostacyclin level was ninefold greater than in fetal PASMC, whereas baseline thromboxane was sevenfold less suggesting greater postnatal cyclooxygenase activity in NBPASMC. PAF decreased, while forskolin and 8‐Br‐cAMP increased cAMP production. Decrease of PAFR effects by Cnucs indicates that normal newborn PA physiology favors vasodilator pathways to minimize PAF‐induced hypertrophy or hyperplasia. We speculate that failure of newborn lung to anchor downregulation of vasoconstrictors with upregulation of vasodilators leads to PPHN.

## Introduction

Platelet‐activating factor (PAF) is a pleiotropic endogenous phospholipid that mediates a diverse range of physiologic and pathologic processes (Montrucchio et al. 2000; Ibe et al. 2002; Stafforini et al. 2003; O'Neill 2005; Liu and Xia 2006; Gao and Raj 2010). It is produced by different cell types, including endothelial cells, smooth muscle cells, and inflammatory cells (Gaumond et al. 1997; O'Neill 2005; Melnikova and Bar‐Eli 2007; Zhou et al. 2007; Tsoupras et al. 2009; Sur and Agrawal 2014). In the lungs, PAF has potent vasoactive effects, acting at different sites in different species (Raj et al. 1992; Toga et al. 1992; Gao and Raj 2010). We have reported that in the fetus, PAF plays an integral physiological role in maintaining high pulmonary vasomotor tone in utero (Ibe et al. 1998). We have also shown that in fetal lamb lungs, PAF receptor (PAFR) binding and *PAFR* mRNA expression are high, whereas in lungs of the newly born lamb, PAFR binding and receptor mRNA expression are low, suggesting a down regulation of PAFR‐mediated effects in vivo (Ibe et al. 1998, 2000). In addition, the effects of PAF can also be completely abrogated by catabolic action of the enzyme PAF acetylhydrolase (Kim et al. 2000), by specific PAF receptor antagonists (Argiolas et al. 1995; Ibe et al. 1998), or by agents that decrease PAF receptor protein expression (Ibe et al. 2000).

PAF generates its effects by binding to its G protein‐coupled receptor (GPCR), which is a seven transmembrane receptor (Parent et al. 1996). Activation of GPCR by an agonist results in the activation of signal transduction pathways (Schoenberg et al. 1999), which may involve recruitment of intracellular second messengers such as cAMP, cGMP, inositol 1,4,5‐triphosphate (IP_3_), and calcium (Lin and Rui 1994; Rehring et al. 1996; Ibe et al. 2007). cGMP and cAMP act via their endogenous receptors, cGMP‐dependent protein kinase (PKG) and cAMP‐dependent protein kinase (PKA), respectively, to elicit relaxation of smooth muscle (Dhanakoti et al. 2000; Abdel‐Latif 2001). We have shown that acute hypoxia upregulates PAF receptor‐mediated intracellular signaling in fetal ovine pulmonary vascular smooth muscle (Ibe et al. 2005). Chronic hypoxia in the perinatal period may result in abnormal upregulation of PAFR protein expression, PAFR binding, and PAFR‐mediated cell signaling (Bixby et al. 2007), leading to an increased pulmonary vasomotor tone and vascular remodeling, key mechanisms contributing to the pathogenesis of persistent pulmonary hypertension of the newborn (PPHN) (Caplan et al. 1990).

Furthermore, the long‐term effects of oxygen therapy on newborn pulmonary vasculature are not completely understood. In the clinical setting, supplemental oxygen has been a traditional mainstay of therapy to treat hypoxic respiratory failure including PPHN. However, there is a growing recognition of the probable injurious effects of hyperoxia and evidence in the literature suggesting the possible mechanisms by which 100% oxygen exposure may potentiate pulmonary vascular dysfunction, even in the short term (Lakshminrusimha et al. 2006, 2007; Farrow et al. 2008, 2012). In vitro studies in ovine fetal pulmonary artery smooth muscle cells (PASMC) have shown that even brief exposure to hyperoxia increases expression and activity of the enzyme phosphodiesterase 5, known to hydrolyze cGMP, thereby shifting the smooth muscle cells toward an imbalance of vasoconstriction (Farrow et al. 2012). Prolonged exposure to supraphysiologic levels of oxygen may therefore counteract the therapeutic effects of inhaled nitric oxide (iNO), used in moderate to severe PPHN for its vasodilatory effects via upregulation of cGMP. This may result in a poor or waning response to iNO and further propagation of pulmonary vascular pathology. The pathologic effects of 100% oxygen were also demonstrated in vivo on neonatal lamb studies when both pulmonary artery contractility and responsiveness to iNO were pathologically affected (Lakshminrusimha et al. 2006, 2007).

We wish to investigate the mechanisms regulating the transition from fetal to newborn pulmonary hemodynamics, a process that is crucial for autonomous oxygenation outside of the uterus. Our primary hypothesis is that with exposure to normoxia at birth and the increased production of prostacyclin, cGMP, and cAMP in pulmonary vascular smooth muscle, PAFR protein expression and PAFR‐mediated cell signaling are downregulated via crosstalk between the cyclic nucleotides and PAF‐PAFR complex. We used newborn ovine intrapulmonary vascular smooth muscle cells to study the effects of cAMP on PAF receptor binding and PAFR‐mediated cell signaling in normoxia, hypoxia, and hyperoxia.

## Materials and Methods

### Materials

The studies were approved by the Institutional Animal Care and Use Committee of the Los Angeles Biomedical Research Institute at Harbor‐UCLA Medical Center. Pregnant ewes (146–148 days gestation, term being 150 days) were purchased from Nebekar Farms, Santa Monica, CA. Authentic standards of PAF hexadecyl‐2‐acetyl‐sn‐glyceryl‐3‐phosphorylcholine (C_16_‐PAF) and lyso‐C_16_‐PAF standards as well as 8‐Br‐cAMP, Rp‐cAMPS were purchased from Biomol, Plymouth Meeting, PA. Radiolabeled PAF standards and substrates; hexadecyl‐2‐acetyl‐sn‐glyceryl‐3‐phosphorylcholine, 1‐O‐[acetyl‐^3^H‐(N)]‐ (^3^H‐acetyl‐C_16_‐PAF), 21.5 Ci/mmol (370 GBq/mmol) were purchased from Perkin Elmer Life Sciences (Boston, MA). Phenylmethysulfonyl fluoride (PMSF), leupeptin, pepstatin, as well as bovine serum albumin (BSA), were purchased from Sigma Chemical Company (St. Louis, MO). Antibody to catalytic domain of PKA (PKA‐C*α*) was purchased from Cell Signaling, while PAF receptor antibody was purchased from Cayman Chemical Company (Ann Arbor, MI). Studies were done with freshly made reagents. Ecolite(+) liquid scintillation cocktail was purchased from ICN Biochemicals (Irvine, CA).

### Methods

#### Study hypothesis

**Figure 1:**
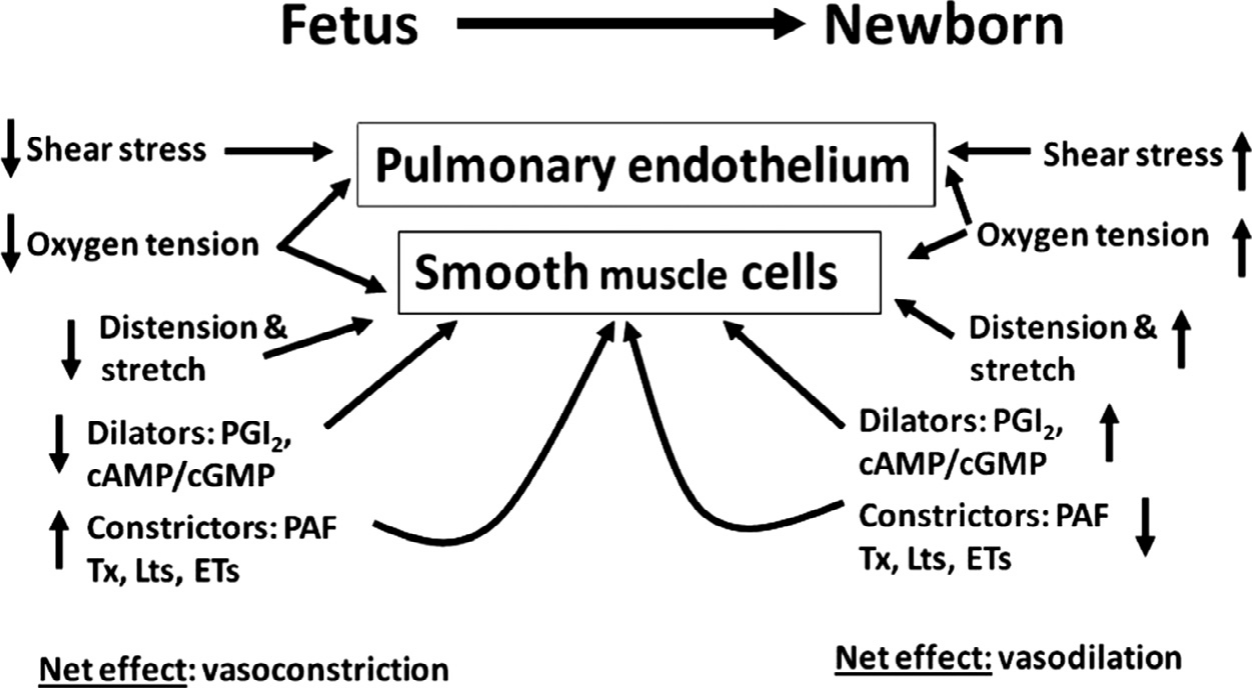
Biochemical and biophysical control of perinatal pulmonary circulation which is summarized from our previous in vivo and in vitro studies in ovine fetal and newborn lambs (Bixby et al. 2007; Heymann et al. 1990; Ibe et al. 1998, 2000, 2002, 2005, 2007) and other perinatal reports (Haworth and Reid 1976; Dhanakoti et al. 2000; Montrucchio et al. 2000). Ovine fetal pulmonary hemodynamics favors vasoconstrictors while newborn hemodynamics favors vasodilators.

Figure 1 shows the normal biophysical and biochemical control of perinatal pulmonary circulation which is summarized from our previous, in vivo and in vitro, studies in ovine fetal and newborn lambs (Ibe et al. 1998, 2000, 2002, 2005, 2007, 2008; Bixby et al. 2007) and from other perinatal reports (Murphy et al. 1981; Heymann et al. 1990; Dhanakoti et al. 2000). Our hypothesis in this study is that the inability of lungs of newborn lambs to downregulate the production and activity of the pulmonary vasoconstrictor such as PAF with concomitant up regulation of production of pulmonary vasodilators such as prostacyclin results in the persistence of the fetal high pulmonary vascular tone. Our strategy in this study involves subjecting the cells in culture to different oxygen tensions: in a 37°C incubator aerated with 5% CO_2_ in air to mimic the normal lung oxygen environment; in an incubator aerated with hypoxia gas mixture to mimic the oxygen environment of fetal lambs; and an incubator aerated with hyperoxia gas mixture to mimic the oxygen environment typically used during PPHN therapy.

#### Preparation of pulmonary arterial smooth muscle cells

Intrapulmonary vessels were isolated from freshly killed 6–12 day old newborn lambs and then smooth muscle cells were harvested from the freshly excised arteries under sterile conditions as previously reported (Ibe et al. 2002; Renteria et al. 2013a). Cells were used at the 4th to 10th passage and identity of the smooth muscle cells at each passage was characterized with an *α*‐smooth muscle cell‐specific monoclonal antibody, catalog #A2547, a myosin light chain kinase proteins catalog #M4401 (105K4806), both from Sigma‐Aldrich, St. Louis, MO. The SMC were devoid of endothelial cells as determined by absence of factor VIII antigen (Stead and Mckee 1979), and fibroblasts by absence of fibroblast‐specific protein 1 (Strutz et al. 1995). Cell phenotype did not change from 4th to 10th passages as determined by the expression of the *α*‐smooth muscle actin and myosin light chain kinase proteins as well as by the SMC morphology.

#### Experimental conditions

All studies were done with adherent cells in normoxia, in hypoxia, or in hyperoxia conditions.

##### Normoxia

Cells were studied in a humidified incubator at 37°C aerated with 5% CO_2_ in air. Incubator and media pO_2_ was 80–100 torr and monitored with TED 60T percent oxygen sensor, Teledyne Analytical Instruments (City of Industry, CA).

##### Hypoxia

An incubator set at 37°C was first equilibrated for at least 1 h with a gas mixture of 2% O_2_, 10% CO_2_, and balance N_2_ to maintain incubator and cell media pO_2_ <40 torr to mimic fetal lung environment, and monitored with TED 60T percent oxygen sensor, Teledyne Analytical Instruments. Cells were then placed in this equilibrated incubator and continuously aerated with the hyperoxia gas mixture throughout the duration of the study (Renteria et al. 2013b).

##### Hyperoxia

An incubator set at 37°C was first equilibrated for at least 1 h with a gas mixture of 95% O_2_, 2.5% CO_2_, and balance N_2_ to maintain incubator and cell media pO_2_ >100 torr and monitored with TED 60T percent oxygen sensor, Teledyne Analytical Instruments. Cells were then placed in this equilibrated incubator and continuously aerated with the hypoxia gas mixture throughout the duration of the study.

### Study of baseline release of prostacyclin and thromboxane by fetal and newborn PASMC in normoxia

We compared baseline production of prostacyclin (PGI_2_) and thromboxane A_2_ by fetal and newborn PASMC to understand the production of this vasodilator and vasoconstrictor by perinatal ovine PASMC in culture. The experiments were set up in parallel in normoxia using fetal PASMC and newborn PASMC. Cells were seeded at 5 × 10^5^ cells in 12‐well culture plates and allowed to attain 80–90% confluence. Then, the cells were washed with protein‐free PBS and incubated for 15 min in normoxia at 37^°^C in Krebs bicarbonate buffer, pH 7.4, containing the following reagents in (mmol/L): NaCl, 119; KCl, 4.6; CaCl_2_, 1.5; MgCl_2_, 1.2; NaHCO_3_, 15; NaH_2_PO_4_, 1.2; glucose, 5.5. After incubation, the media were acidified with 0.1 mL of 10% trichloroacetic acid and aspirated into borosilicate glass tubes and spun at 300 g for 10 min. Prostacyclin as 6‐keto PGF_1*α*_ (6keto) and thromboxane as TxB_2_ were measured from extracted culture media with ELISA kits purchased from Neogen Corporation (Lexington, KY) following the instructions provided by the vendor. The ELISA kits were tested for inter‐ and intraassay variability which were <0.1%. Amounts of 6keto or TxB_2_ measured are presented in nanogram/10^6^cells (ng/10^6^ cells).

### Study of effect of oxygen tension on release of prostacyclin by newborn PASMC

We studied the effect of different oxygen tensions on release of prostacyclin by newborn PASMC. Since we are interested in understanding the role of endogenous PAF in the pulmonary physiology of the newborn lamb, we treated some cells with the 10 *μ*mol/L of the PAFR antagonist CV3988 to inhibit PAF binding and prevent activation of PAFR signaling in the PASMC by the endogenous PAF. We also treated some cells with 10 *μ*mol/L of flurbiprofen, a potent cyclooxygenase inhibitor. Studies were done essentially as described above for the baseline study in normoxia, except that cells were first pulsed for 30 min in the experimental oxygen tension, followed by preincubation for another 30 min with the needed specific inhibitor.

### Study of effect of oxygen tension on cAMP release by newborn lamb pulmonary artery smooth muscle cells

We have reported on cyclic nucleotides production by FPASMC at different oxygen tensions (Ibe et al. 2007), but have not studied the effect of different oxygen tension on cAMP production by newborn PASMC. Therefore, we investigate, in parallel, the effect of normoxia, hypoxia, and hyperoxia on PAF and cAMP stimulation of cAMP production by newborn PASMC. Cells were seeded at 5 × 10^5^ cells in 12‐well culture plates and allowed to attain 80–90% confluence. The subconfluent cells were washed twice with protein‐free PBS and preincubated for 4 h in normoxia, hypoxia, or hyperoxia at 37°C in Krebs bicarbonate buffer, pH 7.4, containing the following reagents in (mmol/L): NaCl, 119; KCl, 4.6; CaCl_2_, 1.5; MgCl_2_, 1.2; NaHCO_3_, 15; NaH_2_PO_4_, 1.2; glucose, 5.5; and 0.1 mmol/L of 3‐isobutyl‐1‐methylxanthine (IBMX), to inhibit spontaneous phosphodiesterase catalyzed breakdown of cAMP produced during experimentation. After preincubation, cells were treated with the following agents: 10 *μ*mol/L of cell‐permeable cAMP analog 8‐Br‐cAMP or 10 nmol/L PAF in one experiment; in a second experiment, cells were treated 10 nmol/L PAF, 0.1 mmol/L forskolin, or both agents concurrently. Treated and control cells were incubated for 15 min more in normoxia, hypoxia, and hyperoxia. After incubation, the media were acidified with 0.1 mL of 10% trichloroacetic acid and aspirated into borosilicate glass tubes. The cells were treated with 0.5 mL of ice‐cold ethanol and the ethanolic cell suspension was combined with its incubation media. Ethanol content was evaporated with nitrogen in ambient temperature. The aqueous fraction was spun for 10 min at 360 g to pellet cell debris. The resulting aqueous supernatant containing the cAMP produced was extracted with diethyl ether saturated with water. The water fraction containing the cyclic nucleotide was lyophilized and then redissolved in 0.05 mol/L sodium acetate which was the cAMP assay buffer supplied by the provider of the EIA assay kit. The amounts of cAMP produced were determined by ELISA with a commercial kit (Biomedical Technologies Inc.) following the instructions provided by the vendor. ELISA inter‐ and intraassay variability were <0.1%. Amounts of cAMP measured are presented as picomol/mL (pmol/mL).

### Study of PAF receptor binding in different oxygen tensions

#### General protocol

We have reported on the effect of hypoxia on PAFR binding to FPASMC, but have studied newborn PASMC during normoxia only. Therefore, we investigate, in parallel, the effect of normoxia, hypoxia, and hyperoxia on PAFR binding to newborn PASMC. Receptor binding assays were performed in conditions of normoxia and hypoxia as previously reported by us (Ibe et al. 2005; Renteria et al. 2010, 2013b), and a third treatment in hyperoxia condition as described above. Briefly, the cells were washed with calcium‐ and magnesium‐free PBS before use according to the specific study protocol. After incubation in normoxia, hypoxia, or hyperoxia, unbound ^3^H‐PAF was washed off with ice‐cold PBS, and then incubated on ice for 30–45 min in saline/EDTA mixture containing 154 mmol/L saline and 5 mmol/L EDTA. Receptor‐bound ^3^H‐PAF was extracted on Whatman GF/C membrane filters using in‐line vacuum system and then subjected to extraction procedures as previously reported (Ibe et al. 2000, 2002, 2005, 2007). Cell‐bound PAF radioactivity was quantified by scintillation spectrometry (Beckman Instruments, Fullerton, CA). In studies probing the interaction of PAF with its receptors in the presence of other agonist or antagonists, cells were preincubated with the agent before the addition of ^3^H‐PAF, and then incubated further according to the specific experimental protocol.

### Specific Protocols

#### PAF receptor (PAFR) binding to membrane proteins

Subconfluent cells in 15 cm Petri dishes were incubated for 24 h in normoxia, hypoxia, or hyperoxia without stimulation, and then cells were washed with ice‐cold PBS and lysed with hypotonic lysis buffer. The lysed cells were spun at 360 g to pellet cell debris, and the 360 g supernatant was spun at 100,000 g for 1 h to harvest the membrane proteins. Membrane proteins were used to assay for PAFR binding at 4°C for 24 h.

#### Effect of acute oxygen tension conditions and specific PAFR and PKA antagonists on PAFR binding

Cells were preincubated for 4 h in hypoxia, normoxia, or hyperoxia with buffer for controls or specific PAFR antagonist 10 *μ*mol/L CV3988 or 10 *μ*mol/L of cAMP‐dependent PKA antagonist Rp‐cAMPS. Then, 1 nmol/L [^3^H]‐PAF was added and incubated for 30 min more in each oxygen condition. PAF bound to receptor was extracted and quantified.

#### Effect of prolonged oxygen tension on PAFR binding

Cells in culture plates were cultured for 72 h in hypoxia, normoxia, or hyperoxia. After 72 h in culture, cells were prepared for study and treated with buffer alone for controls or specific PAFR antagonist 10 *μ*mol/L CV3988 or cAMP‐dependent PKA receptor antagonist, 10 *μ*mol/L Rp‐cAMPS for 30 min. Then, 1 nmol/L [^3^H]‐PAF was added to each treatment condition and incubated for 30 min more. PAF bound to receptor was extracted and quantified.

### Study of stimulation of protein expression at different oxygen tensions

#### Western blotting

##### Preparation of proteins for Western analysis

Proteins were prepared from stimulated and unstimulated cells that were studied in normoxia, hypoxia, or hyperoxia as described above. Briefly, after incubation in normoxia, hypoxia, or hyperoxia, cells were washed with PBS and lysed with a modified 40 mmol/L HEPES hypotonic lysis buffer, pH 7.4, containing the following: 1 mmol/L EGTA, 4 mmol/L EDTA, 2 mmol/L MgCl_2_, 10 mmol/L KCl, 1 mmol/L dithiothreitol (DTT), 0.1 mmol/L PMSF, 5 *μ*g/mL leupeptin, 1 *μ*g/mL pepstatin, 1 *μ*mol/L 4‐(2‐aminoethyl) benzene sulfonyl fluoride, 200 mmol/L sodium fluoride, 20 mmol/L sodium pyrophosphate, 0.2 mmol/L sodium vanadate, and 0.1 mg/mL trypsin inhibitor. Proteins were recovered from lysed cells by centrifugation at 1500 *g* for 15 min in refrigerated Eppendorf bench top centrifuge and stored in 0.2 mL aliquots at −80°C and used for western blotting.

##### Sodium Dodecyl Sulfate‐Polyacrylamide Gel Electropheresis (SDS‐PAGE)

Proteins were subjected to Coomassie blue quantification before use in western blotting with some modification of published methods (Ibe et al. 2000, 2002, 2007). Proteins were suspended in sodium dodecyl sulfate (SDS) sample buffer, pH 6.8, containing: 125 mmol/L Tris‐base, 4% SDS, 0.006% bromophenol blue, 36 mmol/L EDTA, 90 mmol/L DTT, 10% glycerol, 10% beta‐mercaptoethanol. Proteins were loaded on each sample per lane and subjected to SDS‐PAGE gel electrophoresis for 1 h at 200V on 4–12% Tris‐glycine gradient gels (Lonza, Rockland, ME), along with Bio‐Rad kaleidoscope prestained molecular weight markers and protein standards. After 1 h of SDS‐PAGE, proteins were transferred to nitrocellulose membrane by means of mini Trans‐Blot, (Bio‐Rad, Irvine, CA) at 70V and then blocked with 5% nonfat dry milk in 1% T‐TBS overnight. Blots were then incubated for 2–4 with the specific antibody, for instance, 1:500 of PAFR antibody; then, each antibody was washed with 1% T‐TBS, incubated for 1 h with an anti‐rabbit Ig HRP‐linked secondary antibody (Amersham), followed by three more washes with 1% T‐TBS. The signals were developed for 1 min using Amersham ECL Western Blot detection kit and then exposed to X‐ray film. Bands corresponding to PAFR and PKA‐C*α* proteins were scanned and quantified.

### Study of PAF receptor and *PKA‐Cα* gene expression by quantitative reverse transcriptase‐polymerase chain reaction (qRT‐PCR)

RNA from newborn PASMC incubated for 24 h was subjected to qRT‐PCR (qPCR) studies to examine PAF receptor and *PKA‐Cα* gene expression by cells cultured in normoxia, hypoxia, and hyperoxia under baseline conditions and following treatment with an exogenous 10 nmol/L PAF. Total RNA was extracted with Qiagen's QIAshredder (cat #79654) and Rneasy Plus Mini Kit (cat # 74134), according to the manufacturer's protocol (Qiagen, Valencia, CA). Quantitative PCR was performed on ABI Prism 7000 Detection System (Applied Biosystems, Foster City, CA). Reverse transcription was accomplished with Applied Biosystems High Capacity RNA‐cDNA Master Mix (cat # p/n 4390715) program as follows: step 1, 25°C for 5 min; step 2, 42°C for 30 min; step 3, 85°C for 5 min; and step 4, hold at 4°C. The cDNA was subjected to PCR with SYBR‐Green PCR Master Mix (cat # p/n 4309155) as fluorescent dye, with the following steps: step 1, 50°C one cycle; step 2, 95°C 10 min one cycle; step 3, 95°C 15 s, 59°C 1 min, 40 cycles (annealing/amplification); step 4, dissociation cure, one cycle, all done according to the manufacturer's protocol (Applied Biosystems) using primer base pairs (bp) which are: *PAFR* primer #1, forward 5′‐ CCT GTG CAA CGT GGC TGG CT‐3′, bp98‐117, reverse 5′‐ GAG ATG CCA CGC TTG CGG GT‐3′ bp 241‐222 and *PAFR* primer #2, forward 5′– TCC TGT GCA ACG TGG CTG GC‐3′, bp 97‐116, reverse 5′ GAG ATG CCA CGC TTG CGG GT‐3′, bp 241‐222, both created using NCBI's Primer‐BLAST program (http://www.ncbi.nlm.nih.gov.tool/primer-blast/) by entering accession number. The primers for *PKA‐Cα* were as follows: forward 5′‐ CAG AAG GTG AAG CTG AA‐3′, reverse 5′‐ CAG GTC CCG GTA GAT GAG AT‐3′, region bp 242‐514, accession number NM_001009234. All primer sets were authenticated by RealTimePrimers.com. The *GAPDH* primer sequence, GenBank Accession AFO30943 at bp #108‐131 and 399‐375, forward 5′‐ACC TGC CAA CAT CAA GTG GGG TGA T‐3′, reverse 5′‐GGA CAG TGG TCA TAA GTC CCT CCA C‐3′ were synthesized by SIGMA‐Aldrich (Saint Louis, MO). The negative control provided with the RT‐PCR kit was used according to the manufacturer's protocol (Applied Biosystems). The relative quantification in gene expression was calculated using the 2^−∆∆Ct^ method (Livak and Schmittgen 2001) and fold changes were normalized to expression of *GAPDH* internal standard.

### Oxygen tension and cell proliferation

We wished to ascertain that activation of PAFR‐mediated and cAMP/PKA‐mediated signaling can be transduced into the nucleus to affect cell growth and activate PAFR protein expression under our experimental conditions. Therefore, we studied the effect of different oxygen tensions on PAF and cAMP/PKA on cell proliferation. Cells were seeded in 6‐well culture plates at 5 × 10^4^ cells per well and allowed to stabilize for 2–3 days. Cells were then serum‐starved by culturing in 0.1% FBS for 72 h, and then cultured in 10% FBS with or without the test agents in the presence of 5 *μ*Ci/well of ^3^H‐thymidine and incubated for 24 h more in normoxia, hypoxia, or hyperoxia according to the specific protocol. Proliferation assays were done as previously reported (Ibe et al. 2008). Cells labeled with ^3^H‐thymidine were extracted with 0.5 N NaOH. Radioactivity of cell lysate was quantified on LKB 6500 scintillation counter (Beckman Coulter, Fullerton, CA).

### Specific protocols

#### Effect of specific PAFR and PKA antagonists on endogenous PAFR‐ and PKA‐mediated cell proliferation

Effects of specific PAFR antagonist CV3988 and PKA receptor antagonist Rp‐cAMPS on endogenous PAFR‐ and PKA‐ mediated cell proliferation were studied as previously described (Renteria et al. 2013a). Serum‐starved cells were preincubated for 2 h with 10 *μ*mol/L CV3988 or 10 *μ*mol/L Rp‐cAMPS dissolved in 10% FBS growth medium or with 10% FBS growth medium alone, and then 10 nmol/L PAF and 5 *μ*Ci of ^3^H‐thymidine were added and incubated for 24 h more in normoxia, hypoxia, or hyperoxia.

### Data analysis

All numerical data were mean ± SEM. In all instances of radioisotope use, background radioactivity was subtracted before final quantification. Data were analyzed using a two‐tailed *t* test followed by two‐way analysis of variance (ANOVA) with Tukey post hoc test (GraphPad Prism; GraphPad, San Diego, CA). Results were considered significant at *P *<* *0.05.

## Results

### Scheme of research hypothesis

Figure 1: A depiction of the biophysical and biochemical control of perinatal pulmonary circulation which is summarized from our previous, in vivo and in vitro, studies in ovine fetal and newborn lambs (Ibe et al. 1998, 2000, 2002, 2005, 2007, 2008; Bixby et al. 2007) and other perinatal reports (Murphy et al. 1981; Heymann et al. 1990; Dhanakoti et al. 2000). The fetal lung produces predominantly more vasoconstrictors endowing it vasoconstrictor properties. Immediately after birth, the constrictors are downregulated while the dilators are upregulated to endow the lung with vasodilator properties.

### Baseline production of prostacyclin and thromboxane A_2_ by perinatal ovine PASMC

**Figure 2:**
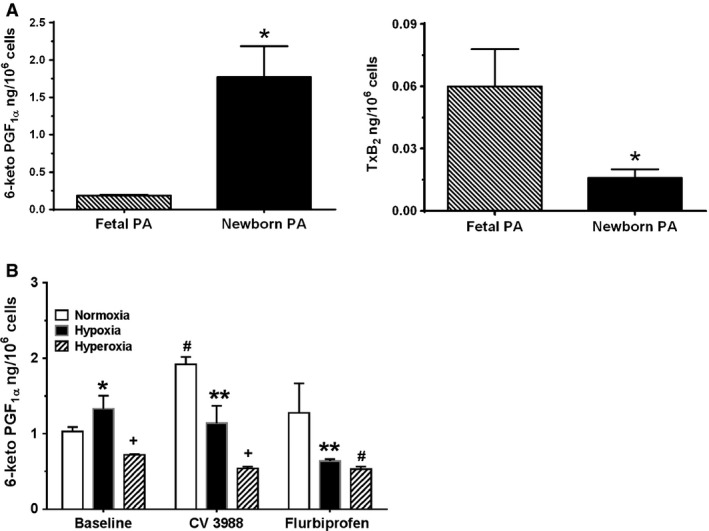
A, Baseline production of prostacyclin and thromboxane by fetal and newborn pulmonary artery smooth muscle cells (PASMC). Release of prostacyclin (PGI2) and thromboxane (TxA2) by fetal and newborn PASMC at baseline was measured by ELISA of their respective stable metabolites 6‐keto‐PGF1*α* and TxB2. Data are mean + SEM, *n* = 6. Production of 6‐keto‐PGF1*α* by newborn PASMC was ninefold greater than by fetal PASMC, whereas baseline production of TxB2 by newborn PA was sevenfold less. *P < 0.05, different from fetal PA. B, Hyperoxia attenuates prostacyclin release by newborn PASMC. Effect of hypoxia and hyperoxia on prostacyclin release was measured as 6‐keto‐PGF1*α*. Data are mean ± SEM, *n* = 6. Hyperoxia attenuated prostacyclin release under all conditions. Inhibition of PAFR with CV3988 increased prostacyclin release. *P < 0.05, different from normoxia; ^+^ *P* < 0.05, different from hypoxia and hyperoxia; **P < 0.05, different from CV3988 or flurbiprofen normoxia; ^#^ *P* < 0.05, different Baseline and CV3988 conditions.

Figure 2A shows baseline production of vasodilator prostacyclin (measured as the inactive metabolite 6‐keto‐PGF_1*α*_) and vasoconstrictor thromboxane A_2_ (measured as the inactive metabolite TxB_2_) by fetal and newborn ovine PASMC, respectively. Baseline production of 6‐keto‐PGF_1*α*_ by newborn PASMC was ninefold greater than by fetal PASMC, whereas baseline production of TxB_2_ by newborn PA was sevenfold less than by fetal PASMC.

### Hyperoxia attenuates PGI_2_ release by newborn PASMC in culture

We then investigated the effect of hypoxia and hyperoxia on prostacyclin release by the newborn PASMC. As shown in Figure 2B, under baseline conditions, there was no difference in prostacyclin production between normoxia and hypoxia. However, hyperoxia conditions attenuated prostacyclin release by 46% compared to normoxia effect. Treatment of the cells with CV3988, to inhibit the effect of endogenous PAF at its receptor led to 46% increase in prostacyclin release in normoxia, suggesting that inhibition of the effect of endogenous PAF at its receptor allowed for more prostacyclin production above the baseline conditions. Effect of CV3988 on prostacyclin release in hypoxia was not different from baseline release in hypoxia. Interestingly, prostacyclin release was decreased threefold in hyperoxia compared to CV3988 effect in normoxia and 50% decrease compared to effect of CV3988 in hypoxia. CV3988 decreased prostacyclin release by 25% compared to hyperoxia effect under baseline conditions. The cyclooxygenase inhibitor, Flurbiprofen, produced no significant difference in prostacyclin release compared to baseline conditions in normoxia, but it decreased prostacyclin release by 50% compared to the effect of Flurbiprofen in normoxia. There was no difference in the effect of Flurbiprofen treatment in hypoxia and hyperoxia, but the release in hyperoxia was significantly less (26% less) than for normoxia under baseline conditions. It is necessary to state that assays of TxA_2_ from these experiments did not yield any useful information. Thromboxane A_2_ release was not among the eicosanoids targeted for study after treatments with different oxygen conditions. Interestingly, the measured amounts were in picogram range and compared favorably with the baseline conditions measured in Figure 2A. This suggests that vasodilator activity of prostacyclin is more important than vasoconstrictor activity of thromboxane A_2_ in smooth muscle cell function in the newborn period.

### Effect of oxygen tension on baseline cAMP release

**Figure 3:**
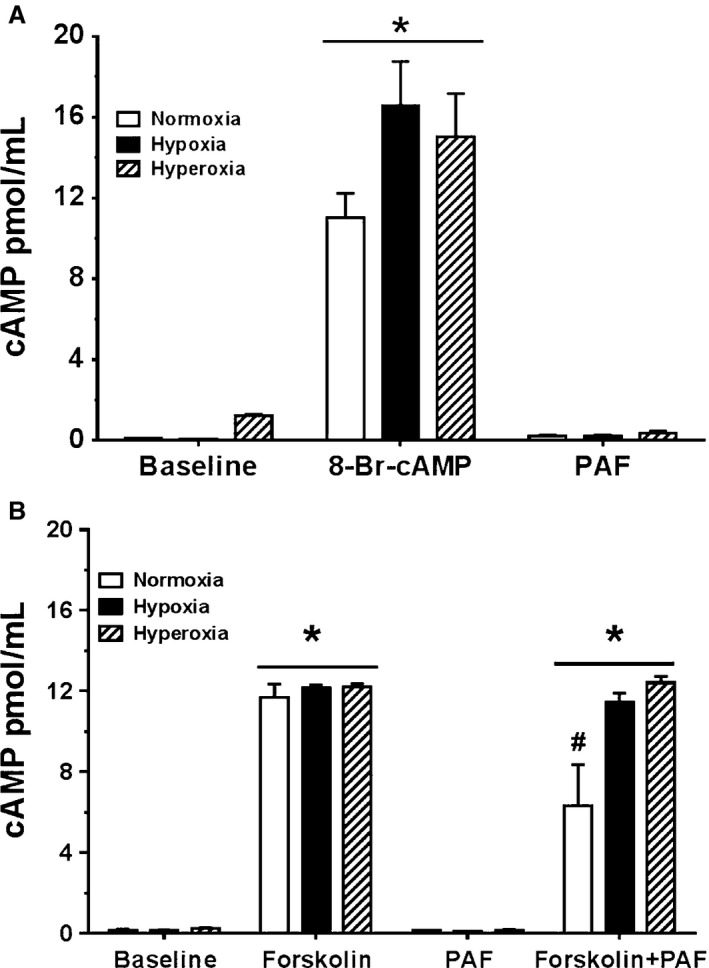
A, Stimulation of cAMP production by newborn pulmonary artery smooth muscle cells (PASMC) subjected to different oxygen tensions, at baseline and treated with exogenous platelet‐activating factor (PAF) and cAMP analog. Release of cAMP was measured by ELISA as described in methods. Data are mean ± SEM, *n* = 6. Cells were seeded at 5 × 10^5^ cells in 12‐well culture plates and allowed to attain 80–90% confluence. Cells were preincubated for 4 h in normoxia, hypoxia, and hyperoxia and treated with 10 nmol/L PAF or 10 *μ*mol/L of cell permeable cAMP analog 8‐Br‐cAMP, then incubated for 15 min more in normoxia, hypoxia, and hyperoxia. Unstimulated newborn PASMC yielded no significant release of cAMP. Stimulation with 8‐Br‐cAMP created significant release of cAMP (11.0 pmol/mL in normoxia, 16.5 pmol/mL in hypoxia and 15.0 pmol/mL in hyperoxia). Treatment with physiologic equivalent of exogenous PAF 10 nmol/L stimulated no significant release of cAMP. *P < 0.05, different from baseline. B, Stimulation of cAMP production by newborn PASMC subjected to different oxygen tensions, at baseline and treated with exogenous PAF and forskolin. Release of cAMP was measured by ELISA as described in methods. Data are mean ± SEM,* n* = 6. Cells were seeded at 5 × 10^5^ cells in 12‐well culture plates and allowed to attain 80–90% confluence. Cells were preincubated for 4 h in normoxia, hypoxia, and hyperoxia, treated with the following agents; 10 nmol/L PAF, and 0.1 mmol/L each of forskolin and both agents concurrently and incubated for 15 min more in normoxia, hypoxia, and hyperoxia. Unstimulated NBPASMC yielded no significant release of cAMP. Stimulation with forskolin created significant release of cAMP. Treatment with physiologic equivalent of exogenous PAF 10 nmol/L stimulated no significant release of cAMP. Cotreatment with both forskolin and PAF resulted in cAMP release, either equivalent to levels following stimulation by forskolin alone, or decreased cAMP release by 46% in normoxia. *P < 0.05, different from baseline; ^#^ *P* < 0.05, different from forskolin treatment alone.

Figure 3A demonstrates cAMP release by newborn PASMC at baseline without stimulation, and following stimulation with cAMP analog 8‐Br‐cAMP or with PAF. Under baseline unstimulated conditions, cAMP release by cells in normoxia and hypoxia was negligible, 0.116 ± 0.006 and 0.07 ± 0.007 pmol cAMP/ml, respectively, but release in baseline hyperoxia was 10‐fold higher compared to release in these baseline normoxia or hypoxia. Stimulation with 8‐Br‐cAMP led to a significant release of cAMP (11.0 pmol/mL in normoxia, 16.5 pmol/mL in hypoxia, and 15.0 pmol/mL in hyperoxia). cAMP release by 8‐Br‐cAMP‐treated cells in hypoxia and hyperoxia was significantly more than in normoxia, but there was no difference in release between hypoxia and hyperoxia. Treatment of cells with physiologic equivalent of exogenous PAF, 10 nmol/L, stimulated 0.21 pmol cAMP in normoxia with no difference with hypoxia, however, release of cAMP by PAF treated cells in hyperoxia was 44% greater than release in normoxia or hypoxia.

### Effect of forskolin on cAMP release

In Figure 3B, we show a consistent effect of baseline conditions on cAMP release by the PASMC similar to Figure 3A, (0.14 pmol/mL in normoxia, 0.16 pmol/mL in hypoxia, and about twofold increase in hyperoxia) compared to normoxia or hypoxia. Stimulation with forskolin, a general direct activator of adenylyl cyclase, created significant release of cAMP (11.7 pmol/mL in normoxia, 12.2 pmol/mL in hypoxia, and 12.2 pmol/mL in hyperoxia). Treatment with physiologic equivalent of exogenous PAF 10 nmol/L produced no significant release of cAMP as was observed in Figure 3A. Finally, cotreatment with both forskolin and PAF resulted in cAMP release in all three conditions. In this condition, co‐incubation of forskolin and PAF decreased cAMP release by 46% in normoxia, but there was no difference in cAMP release by co‐incubation of forskolin and PAF in hypoxia and hyperoxia compared to forskolin alone.

### Effect of oxygen tension on PAF receptor binding to membrane protein

**Figure 4:**
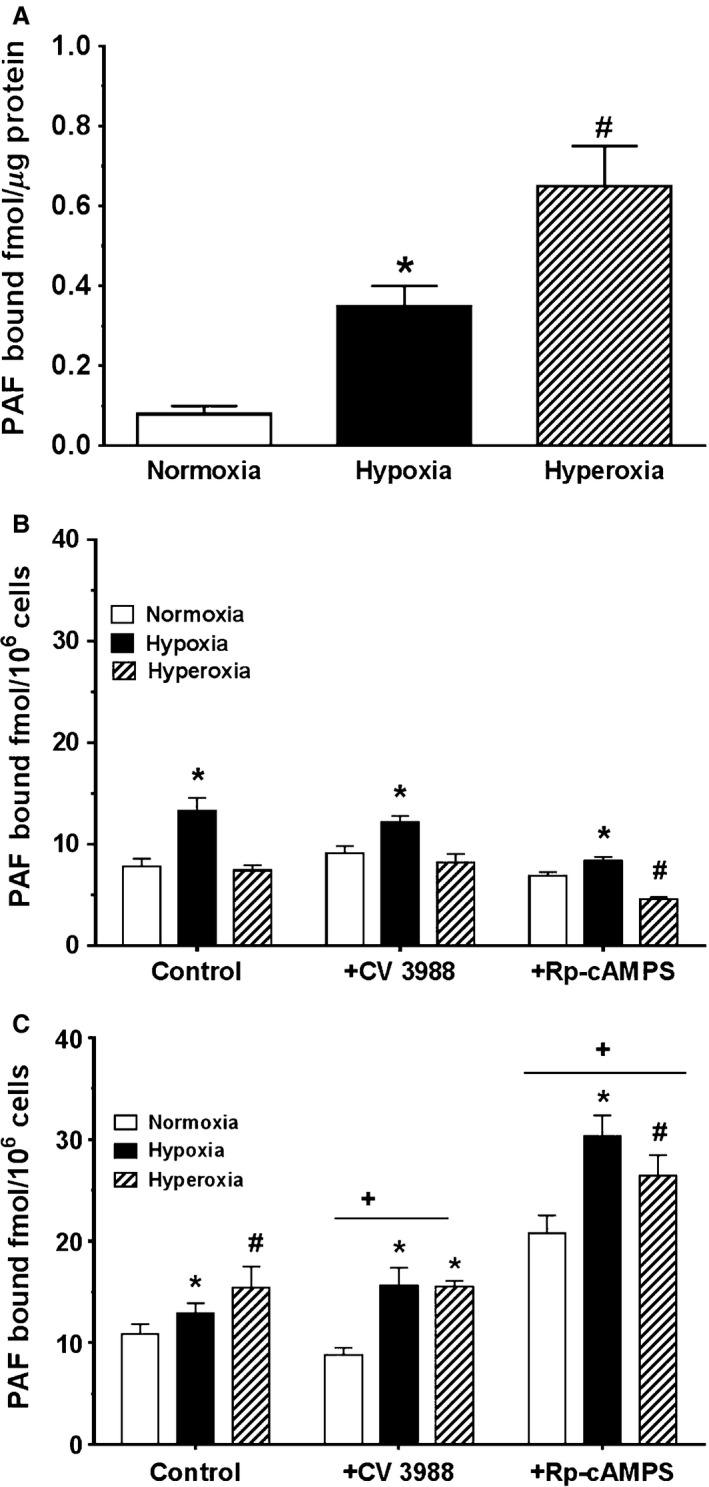
A, Baseline effect of normoxia, hypoxia, and hyperoxia on platelet‐activating factor (PAF) receptor binding in newborn pulmonary artery smooth muscle cells (PASMC) membrane proteins. Membrane proteins were isolated from cells cultured for 24 h in normoxia, hypoxia, or hyperoxia and subject to ^3^H‐PAF ligand binding as described in methods. Data are mean ± SEM,* n* = 4 for each oxygen tensions. PAF binding in normoxia was 0.08 fmol/microgram of membrane protein. Hypoxia increased binding over fourfold more than normoxia, and hyperoxia increased binding eightfold more than normoxia. *p < 0.05, different from normoxia, ^#^ *P* < 0.05, different from normoxia or hypoxia. B, Effect of acute exposure to oxygen tension on PAFR binding to adherent newborn PASMC. Adherent newborn PASMC were preincubated for 4 h in normoxia, hypoxia, and hyperoxia, then subjected to ^3^H‐PAF ligand binding as described in methods. Data are mean ± SEM,* n* = 4 for each oxygen tensions. Baseline‐specific PAF receptor binding to adherent cells following 4 h incubation in normoxia was 7.85 ± 0.77 fmol/10^6^cells. Hypoxia augmented specific PAF receptor binding by 70% but no significant difference was demonstrated following 4 h incubation in hyperoxia. *P < 0.05, different from normoxia, ^#^ *P* < 0.05, different from normoxia or hypoxia. C, Effect of prolonged exposure to different oxygen tension on PAF receptor binding to newborn PASMC. Adherent newborn PASMC were cultured for 72 h in normoxia, hypoxia, and hyperoxia, then subjected to ^3^H‐PAF ligand binding as described in methods. Data are mean ± SEM,* n* = 4 for each oxygen tension. In general, prolonged hypoxia or hyperoxia increased PAFR binding to adherent cells. Incubation for 72 h in hypoxia produced a 19% increase in binding in comparison with normoxia. Incubation in 72 h of hyperoxia amplified binding by over 40% compared to 72 h of normoxia. Pretreatment with CV3988 decreased binding in normoxia but increased binding in hypoxia in comparison with controls. Pretreatment with Rp‐cAMPS significantly increased specific PAFR binding in all three oxygen conditions, a twofold increase in binding in normoxia, 1.5‐fold increase in binding in hypoxia, and 70% increase in binding in hyperoxia. *P < 0.05, different from normoxia; ^#^ *P* < 0.05, different from normoxia or hypoxia; ^+^ *P* < 0.05, different from control and CV3988 conditions.

Since we are interested in understanding the role of PAF‐PAFR‐mediated mechanisms in perinatal pulmonary hemodynamics and the condition of PPHN, we studied PAF binding to its receptors in the newborn PASMC. In Figure 4A, we show the effect of different oxygen tensions on specific PAFR binding to newborn PASMC membrane proteins prepared from cells cultured for 24 h in normoxia, hypoxia, and hyperoxia without stimulation. PAF binding in normoxia was 0.08 fmol/microgram of membrane protein. Compared to normoxia conditions, binding was increased over fourfold in membrane protein prepared from 24 h culture hypoxia compared to binding to membrane proteins from cells cultured in 24 h normoxia. Also, 24 h of hyperoxia increased binding eightfold over normoxia conditions and twofold over hypoxia conditions, thereby indicating different oxygen tension effects on PAFR protein expression by the newborn PASMC.

### Effect of 4 h hypoxia and hyperoxia on PAF receptor binding to adherent cells

Figure 4B shows the effect of normoxia, hypoxia, and hyperoxia on PAF receptor binding to cells incubated for 4 h in normoxia, hypoxia, and hyperoxia, and then PAFR binding was measured utilizing protocol for adherent cells. Baseline‐specific PAFR binding to adherent cells following the 4 h incubation in normoxia was 7.85 ± 0.77 fmol/10^6^ cells. Hypoxia augmented specific PAFR binding by 70% compared to baseline (control) conditions, but no significant difference was demonstrated following 4 h incubation in hyperoxia compared to normoxia. Pretreatment of cells with the PAF receptor antagonist CV3988 increased PAFR binding by 14% compared to control conditions in normoxia. Hypoxia also augmented PAFR binding to CV3988‐treated cells compared to its normoxia counterpart, but produced no difference in binding to CV3988 cells in hyperoxia compared to the normoxia conditions. Compared to control conditions, treatment of cells with Rp‐cAMPS attenuated PAFR binding to the cell from the three oxygen conditions, even though binding in hypoxia was still higher as observed with control and CV3988‐treated cells.

### Prolonged hyperoxia modulates PAF receptor binding to adherent cells

Figure 4C demonstrates the results of PAF receptor binding assays repeated under prolonged incubation (72 h) in normoxia, hypoxia, and hyperoxia, utilizing protocol for adherent cells. Specific PAFR binding to adherent cells following the 72 h culture in normoxia was 10.84 ± 1.02 fmol/10^6^cells which is about 30% increase in binding compared to 4 h culture conditions. Incubation in hypoxia produced a significant 19% increase in PAFR binding over normoxia condition, while incubation in prolonged hyperoxia amplified binding by over 40% compared to 72 h normoxia condition. Thus, prolonged incubation in the respective oxygen conditions was sufficient to induce PAFR protein expression which was not observed in the 4 h incubation. In general, except for 72 h condition of hypoxia, PAFR binding to unstimulated control cells after 72 h in different oxygen tensions was significantly greater than binding after 4 h in the different oxygen conditions, Figure 4B. Pretreatment of cells with CV3988, a PAFR antagonist, decreased binding in normoxia but increased binding in hypoxia in comparison with control conditions. No significant difference was seen with CV3988 pretreatment in hypoxia and hyperoxia. Pretreatment with cAMP/PKA antagonist Rp‐cAMPS resulted in significantly increased specific PAFR binding in all three oxygen tension conditions over their respective controls, suggesting a role of cAMP/PKA in counteracting PAF receptor‐mediated signaling. After 72 h culture in normoxia, hypoxia, and hyperoxia, pretreatment with Rp‐cAMPS resulted in a twofold increase in binding in normoxia, 1.5‐fold increase in binding after 72 h hypoxia, and 70% increase in binding after 72 h culture in hyperoxia, all compared to their respective control conditions.

### Effect of normoxia, hypoxia, and hyperoxia and the cyclic nucleotides cAMP and cGMP on PAFR protein expression under baseline and stimulated conditions

**Figure 5:**
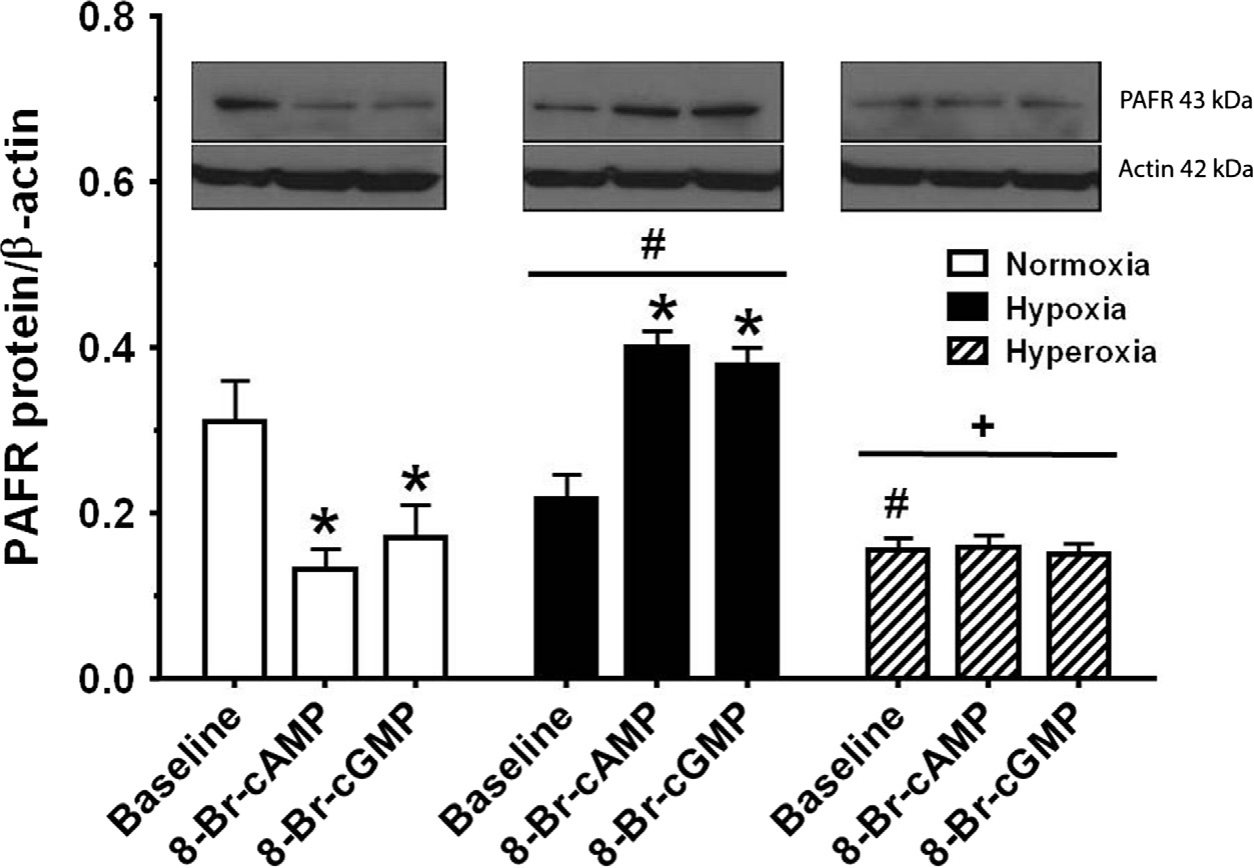
Oxygen tension effect on platelet‐activating factor (PAF) receptor protein expression, studied in control conditions and treatments with cAMP/cGMP analogs. Western blotting was performed on the same gel as described in methods. Receptor protein expression was quantified by protein loading, normalized to total actin expression. Data are mean ± SEM, *n* = 3 different determinations. At baseline, hypoxia and hyperoxia decreased PAFR expression from normoxia control. In normoxia, treatment with cAMP and cGMP analogs both attenuated PAFR expression, while under hypoxic conditions treatment with cAMP and cGMP analogs augmented PAFR expression. In hyperoxic conditions, no difference was observed among treated and untreated cells. Statistics are: *P < 0.05, different from baseline; ^#^ *P* < 0.05, different from normoxia; ^+^ *P* < 0.05, different from hypoxia.

**Figure 6:**
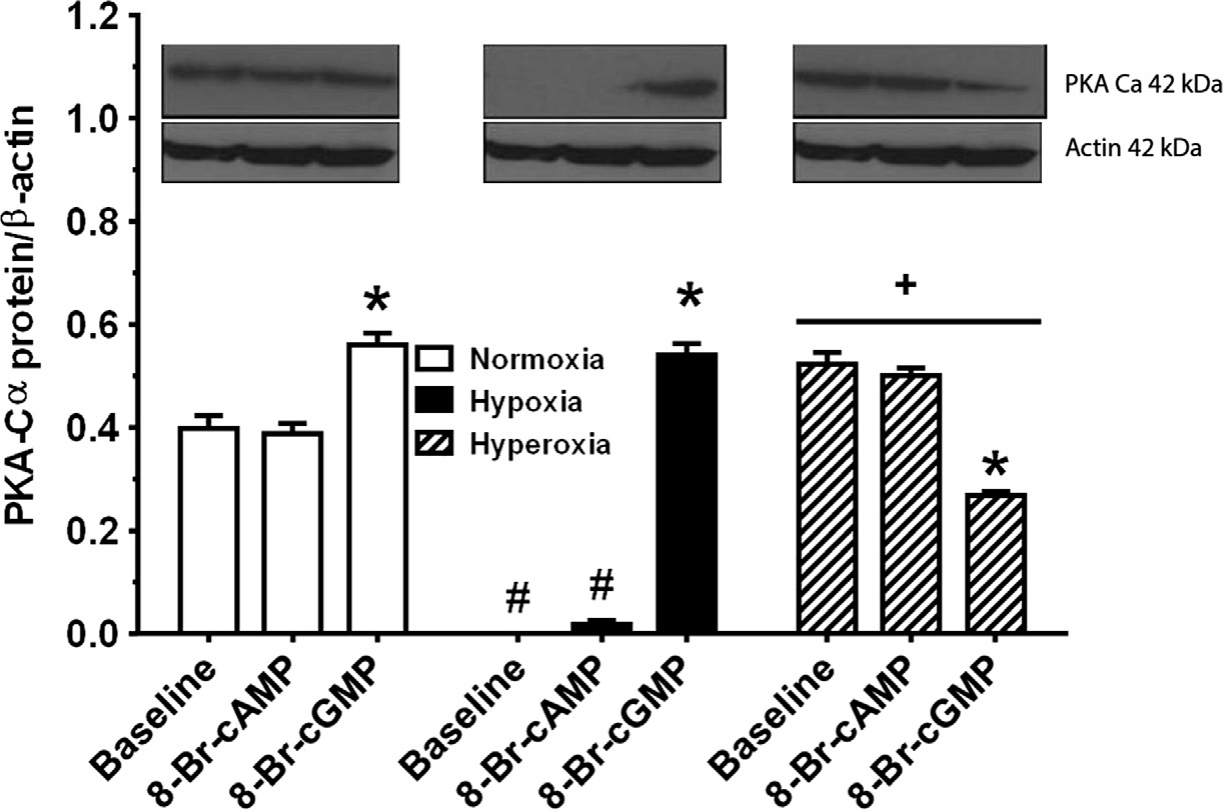
Oxygen tension effect on PKA‐C*α* protein expression, studied in controls and treatments with cAMP/cGMP analogs. Western blotting was performed on the same gel as described in methods. Receptor protein expression was quantified by protein loading, normalized to total actin expression. Data are mean ± SEM,* n* = 3 different determinations. In normoxia, PKA‐C*α* expression was the same in untreated cells and cells treated with cAMP analog, but treatment with cGMP analog augmented PKA‐C*α* expression. Hypoxia completely obliterated the expression of PKA‐C*α* protein and with cyclic nucleotide treatment, only cGMP analog rescued expression of PKA‐C*α*, while treatment with cAMP analog resulted in the minimal expression of PKA‐C*α*. Hyperoxia increased PKA‐C*α* expression over normoxia with no difference observed with cAMP analog treatment. In hyperoxia, treatment with cGMP analog decreased PKA‐C*α* expression. *P < 0.05, different from Baseline, ^#^ *P* < 0.05, different from normoxia, ^+^ *P* < 0.05, different from normoxia or hypoxia.

We have shown crosstalk between cyclic nucleotides and PAF‐PAFR signaling in ovine fetal PASMC (Ibe et al. 2007). We examined the existence of this crosstalk in newborn PASMC. Figure 5 shows the western blotting of PAFR protein expression by newborn PASMC under all three oxygen tension conditions as well as following treatment of cells with membrane‐permeable cAMP and cGMP analogs, respectively. The protein samples were run on the same gel and probed for the PAFR and PKA‐C*α* as well as for actin standard. However, it was confusing when the quantification data were plotted as shown in the rest of the figures (Figs. 2B–4C). To eliminate the confusion, it was necessary to cut the blots and plot the data with the corresponding oxygen conditions as shown in Figures 5, 6. Receptor protein expression was quantified by protein loading and normalized to actin expression. In normoxia, treatment with membrane‐permeable cAMP and cGMP analogs both attenuated PAFR expression, a finding consistent with that previously demonstrated in fetal PASMC exposed to both normoxic and hypoxic conditions (Ibe et al. 2007). In hypoxia, treatment with cyclic nucleotides increased PAFR protein expression which was different from observations in normoxia. Treatment of newborn PASMC with membrane‐permeable cAMP and cGMP analogs augmented PAFR expression in hypoxia, while in hyperoxic conditions, PAFR protein expression was generally decreased compared to baseline expression in normoxia.

### Effect of normoxia, hypoxia, and hyperoxia and the cyclic nucleotides cAMP and cGMP on PKA‐C*α* protein expression under baseline and stimulated conditions

Figure 6 shows western blotting of PKA‐C*α* protein expression by newborn PASMC under all the three oxygen tension conditions as well as following treatment of cells with membrane‐permeable cAMP and cGMP analogs, respectively. PKA‐C*α* protein expression was quantified by protein loading and normalized to actin expression. In normoxia, PKA‐C*α* expression was the same as in untreated cells and cells treated with cAMP analog. Treatment with cGMP analog in normoxia, however, augmented PKA‐C*α* expression. Hypoxia completely obliterated expression of PKA‐C*α* protein under baseline conditions. Treatment with cAMP analog resulted in minimal expression PKA‐C*α*, while treatment with cGMP analog completely restored expression of PKA‐C*α* to its level in normoxia conditions. Hyperoxia increased PKA‐C*α* expression in baseline conditions compared to baseline normoxia and hypoxia. Treatment with cAMP analog also increased PKA‐C*α* expression compared to expression in normoxia and hypoxia, but expression was not different from expression in baseline conditions in hyperoxia. Of note, in hyperoxia treatment with cGMP analog resulted in the opposite effect than it had under conditions of normoxia and hypoxia – it decreased PKA‐C*α* expression rather than increasing it.

### Oxygen tension effect on *PAFR* gene expression in baseline and on stimulation with PAF

**Figure 7:**
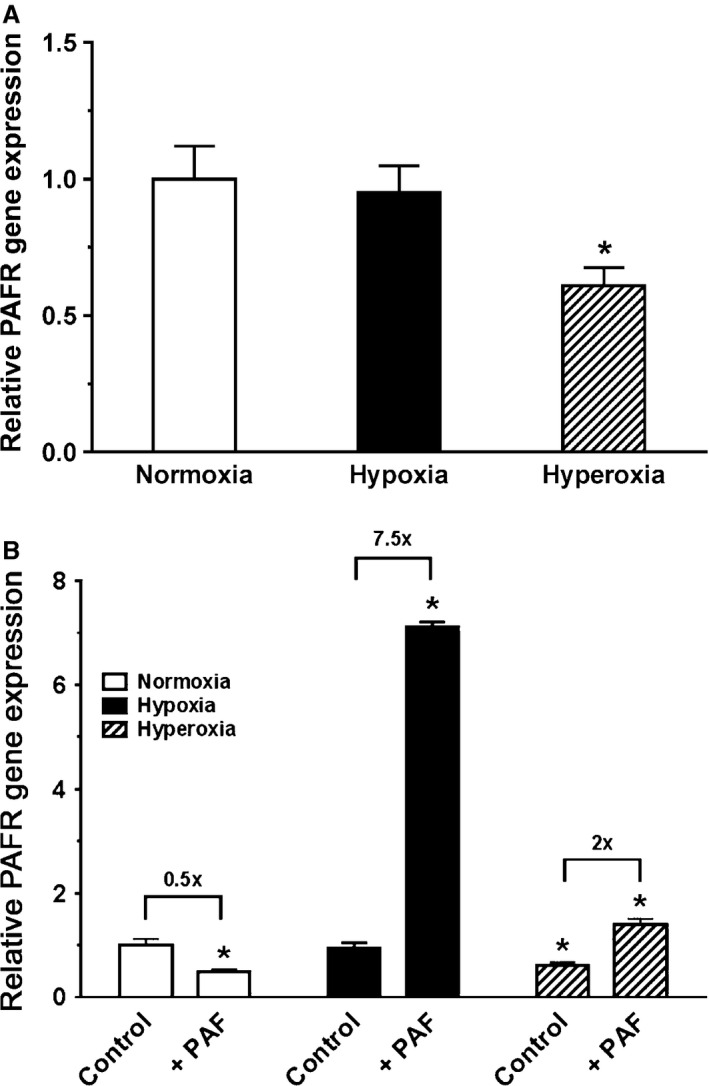
A, Oxygen tension effect on *PAFR* gene expression, studied by qRT‐PCR in unstimulated newborn pulmonary artery smooth muscle cells (PASMC). RNA from newborn PASMC was studied by quantitative RT‐PCR (qRT‐PCR) studies to examine platelet‐activating factor (PAF) receptor expression by cells cultured in normoxia, hypoxia, and hyperoxia under baseline conditions. Gene expression was normalized to expression of *GAPDH* internal standard. Data are mean ± SEM,* n* = 3 different cell preparations. At baseline, exposure to hypoxia made no significant difference in *PAFR* gene expression over normoxia, but hyperoxia exposure decreased *PAFR* expression by 40%. The statistics are: *P < 0.05, different from normoxia. B, Oxygen tension effect on *PAFR* gene expression, studied by qRT‐PCR in controls and treatments with PAF. *PAFR* gene expression by unstimulated controls from Figure 7A which are presented here in comparison with newborn PASMC that were concurrently stimulated with exogenous 10 nmol/L PAF. All values were calculated relative to untreated normoxia‐exposed controls. Ratios of gene expression in untreated to treated cells were calculated within each oxygen tension and presented in fold difference above brackets. Gene expression was normalized to expression of *GAPDH* internal standard. Data are mean ± SEM,* n* = 3 different cell preparations. The greatest *PAFR* gene expression was from PAF‐stimulated cells in hypoxia (seven times greater than unstimulated controls in normoxia). *PAFR* gene expression was also significantly increased in PAF‐stimulated cells exposed to hyperoxia (27% greater than unstimulated controls in normoxia). Stimulation with exogenous PAF decreased *PAFR* gene expression in normoxia by 50%. PAF stimulation increased *PAFR* gene expression in hypoxia‐exposed cells 7.5‐fold and doubled it in hyperoxia‐exposed cells. *P < 0.05, different from normoxia‐exposed untreated controls.

Figure 7A illustrates oxygen tension effect on *PAFR* gene expression, studied by qRT‐PCR in unstimulated newborn PASMC cultured in normoxia, hypoxia, and hyperoxia under baseline conditions. Gene expression was normalized to expression of *GAPDH* internal standard. At baseline, exposure of cells to hypoxia for 24 h produced no significant difference in *PAFR* gene expression over normoxia, but exposure of cells to hyperoxia decreased *PAFR* gene expression by 40%. In Figure 7B, we show the results of *PAFR* gene expression in unstimulated controls from Figure 7A presented in comparison with cells that were concurrently stimulated with exogenous 10 nmol/L PAF. All values were calculated relative to untreated cells exposed to controls in normoxia and gene expression was normalized to expression of *GAPDH* internal standard. Treatment of cells with PAF in hypoxia produced the greatest *PAFR* gene expression (seven times greater than unstimulated controls in normoxia or hypoxia). *PAFR* gene expression was also significantly increased in PAF‐stimulated cells exposed to hyperoxia, 27% greater than unstimulated controls in normoxia, but twofold increase compared to control cells in hyperoxia. Stimulation with exogenous PAF decreased *PAFR* gene expression in normoxia by 50%. However, PAF stimulation increased *PAFR* gene expression in hypoxia‐exposed cells 7.5‐fold and doubled it in hyperoxia‐exposed cells, showing that PAF has autocoid effects – it is a ligand with the ability to upregulate gene expression of its own receptor.

### Oxygen tension effect on *PKA‐Cα* gene expression in baseline and on stimulation with PAF

**Figure 8:**
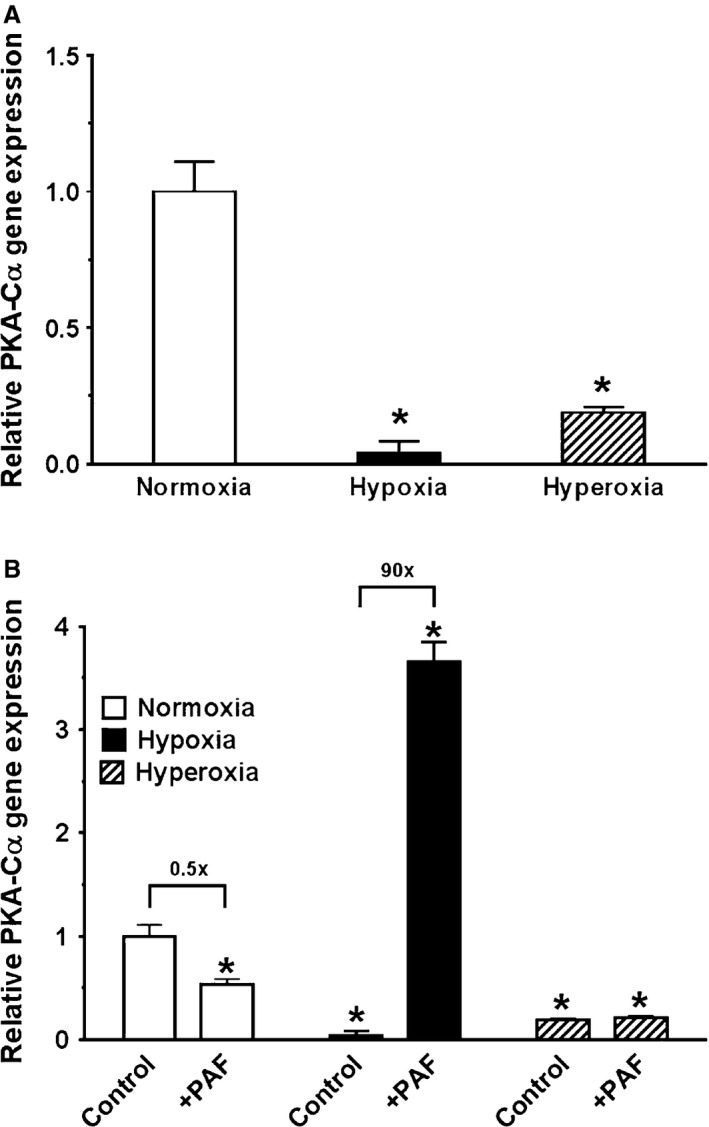
A, Oxygen tension effect on *PKA‐Cα* gene expression, studied by qRT‐PCR in unstimulated newborn pulmonary artery smooth muscle cells (PASMC). RNA from newborn PASMC was studied by quantitative RT‐PCR (qRT‐PCR) studies to examine *PKA‐Cα* gene expression by cells cultured in normoxia, hypoxia, and hyperoxia under baseline conditions. Gene expression was normalized to expression of *GAPDH* internal standard. Data are mean ± SEM,* n* = 3 different cell preparations. Both exposure to hypoxia and hyperoxia decreased *PKA‐Cα* gene expression significantly (hypoxia by 96% and hyperoxia by 81%, respectively). The statistics are: *P < 0.05, different from normoxia. B, Oxygen tension effect on *PKA‐Cα* gene expression, studied by qRT‐PCR in controls and treatments with platelet‐activating factor (PAF). The results of *PKA‐Cα* gene expression in unstimulated controls from Figure 6a are presented here in comparison with newborn PASMC that were concurrently stimulated with exogenous 10 nmol/L PAF. All values were calculated relative to untreated normoxia‐exposed controls. Ratios of gene expression in untreated to treated cells were calculated within each oxygen tension and presented in fold difference above brackets. Gene expression was normalized to expression of *GAPDH* internal standard. Data are mean ± SEM,* n* = 3 different cell preparations. The greatest *PKA‐Cα* gene expression yielded from PAF‐stimulated cells in hypoxia (3.6 times greater than unstimulated controls in normoxia). *PKA‐Cα* gene expression was significantly decreased by hyperoxia exposure, regardless of treatment with PAF. Stimulation with exogenous PAF decreased *PKA‐Cα* gene expression in normoxia by 50%. The statistics are: *P < 0.05, different from normoxia‐exposed untreated controls.

Figure 8A demonstrates oxygen tension effect on *PKA‐Cα* gene expression, studied by qRT‐PCR in unstimulated newborn PASMC cultured in normoxia, hypoxia, and hyperoxia under baseline conditions. Gene expression was normalized to expression of *GAPDH* internal standard. Exposure to hypoxia and hyperoxia significantly decreased *PKA‐Cα* gene expression (hypoxia by 96% and hyperoxia by 81%, respectively). Figure 8B illustrates the results of *PKA‐Cα* gene expression in unstimulated controls from Figure 8A presented in comparison with newborn PASMC that were concurrently stimulated with exogenous 10 nmol/L PAF. All values were calculated relative to untreated cells exposed to normoxia conditions (control), and gene expression was normalized to expression of *GAPDH* internal standard. The greatest *PKA‐Cα* gene expression was by PAF‐stimulated cells in hypoxia (3.6 times greater than unstimulated controls in normoxia). *PKA‐Cα* gene expression was significantly decreased by hyperoxia exposure, regardless of treatment with PAF. Stimulation with exogenous PAF decreased *PKA‐Cα* gene expression in normoxia by 50%.

### PAF stimulation of newborn PASMC proliferation is via PAFR signaling

**Figure 9:**
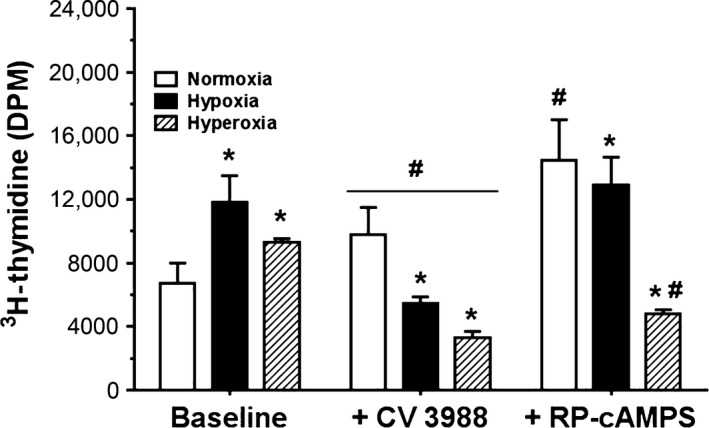
Oxygen tension, specific PAFR antagonist CV3988, and PKA antagonist Rp‐cAMPS effects on endogenous PAFR‐ and PKA‐mediated cell proliferation. Serum‐starved cells were preincubated for 2 h in 10% FBS growth medium without additive, with dissolved 10 *μ*mol/L CV3988 or 10 *μ*mol/L Rp‐cAMPS, respectively, followed by the addition of 10 nmol/L platelet‐activating factor (PAF) and 5 *μ*Ci of ^3^H‐thymidine and 24 h incubations in normoxia, hypoxia, or hyperoxia. Proliferation was quantified as ^3^H‐thymidine uptake in disintegrations per minute (DPM). Data are mean ± SEM,* n* = 6. Hypoxia increased newborn pulmonary artery smooth muscle cells (PASMC) proliferation over normoxia by 72%, while hyperoxia increased newborn PASMC proliferation by 25%. In normoxia, CV3988 increased proliferation; however, in hypoxia and hyperoxia, pretreatment with CV3988 attenuated newborn PASMC proliferation. In normoxia, Rp‐cAMPS enhanced proliferation, while in hypoxia, pretreatment with Rp‐cAMPS made no difference in proliferation and in hyperoxia, it decreased proliferation. The statistics are: *P < 0.05, different from normoxia; ^#^ *p* < 0.05, different from control in each oxygen tension.

Figure 9 illustrates the effects of oxygen tension on newborn PASMC proliferation in baseline conditions and on treatment with CV3988 and Rp‐cAMPS. Under baseline conditions, hypoxia increased newborn PASMC proliferation over normoxia by 43%, while hyperoxia increased PASMC proliferation by 26% over baseline normoxia effect. Figure 9 also demonstrates the effects of treatment of PAFR and PKA antagonists on newborn PASMC within conditions of normoxia, hypoxia, and hyperoxia, respectively. To investigate endogenous PAFR‐mediation of newborn PASMC proliferation within the three oxygen tensions, we measured proliferation following pretreatment with the specific PAFR antagonist CV3988. In normoxia, CV3988 increased proliferation, consistent with our findings in previous studies in which CV3988 augmented proliferation in newborn PASMC but inhibited proliferation in fetal PASMC (Renteria et al. 2013b). However, while this was the case in normoxic conditions, pretreatment with CV3988 under conditions of hypoxia and hyperoxia did attenuate newborn PASMC proliferation. This indicates the involvement of endogenous PAF‐PAFR interactions to promote proliferation in newborn cells only in abnormal conditions. We further investigated the effect of pretreatment of the cells with the PKA antagonist Rp‐cAMPS to inhibit endogenous cAMP‐PKA effect on cell signaling. In normoxia, Rp‐cAMPS enhanced proliferation even greater than baseline condition in normoxia, indicating a role for endogenous cAMP/PKA in homeostatic cell growth under normoxic conditions. In hypoxia, pretreatment with Rp‐cAMPS also increased cell proliferation compared to baseline normoxia condition, but with no difference in the effect in baseline hypoxia. Treatment of cells with Rp‐cAMPS in hyperoxia significantly decreased proliferation compared to effect in baseline hyperoxia.

## Discussion

Various factors including endogenous and exogenous vasoactive agents regulate cell proliferation and the high myogenic tone that are vital to normal intrauterine pulmonary development and physiology. Likely, the most dramatic process in animal physiology is the hemodynamic transition of the lungs from intrauterine vasoconstriction to postnatal vasodilation, allowing for its critical function of gas exchange after birth, that require striking shifts in regulatory signaling cascades. Here, we demonstrate for the first time, in an in vitro model of newborn ovine PASMC, the oxygen tension sensitivity of PAF receptor binding and its downstream effects. Most notably, we show the capacity of not only hypoxia but that of prolonged hyperoxia for PAF‐directed cellular mechanistic derangements implicated in the pathogenesis of PPHN.

### Hyperoxia and prostacyclin release

We opened this study by determining the basal levels of vasodilator metabolite prostacyclin (PGI_2_) and vasoconstrictor TxA_2_, respectively, generated by newborn PASMC in comparison with that by fetal PASMC. At baseline, newborn PASMC baseline production of PGI_2_ was ninefold greater than by fetal PASMC, whereas baseline production of TxA_2_ by newborn PASMC was sevenfold less than fetal production, Figure 2A. These in vitro findings corroborate the discrete temporal switch in perinatal lung physiology from intrauterine vasoconstriction to postnatal vasodilation, when the high intrinsic tone of fetal pulmonary circulation undergoes a fundamental downregulation following birth. In further testing, we found that while hypoxia augmented prostacyclin release, hyperoxia attenuated prostacyclin release. Interestingly, inhibition of endogenous PAF receptor activation with CV3988 increased prostacyclin release, suggesting a link between PAFR‐mediated signaling and cyclooxygenase (cAMP/PKA) activity which as expected was inhibited by Flurbiprofen. Also, since prostacyclin release was attenuated by hyperoxia under the three oxygen tension study conditions, it would seem that PPHN conditions may be enhanced under hyperoxia therapy. Our assays of thromboxane A_2_ production by these cells, to compare with Figure 2B, did not produce any differences in release among the conditions (data not shown), even though the production was significantly less than prostacyclin production. This suggests that postnatal production of vasodilators is more important than production of vasoconstrictor under nonpathological conditions. The relevance of this finding is not clear at this time and does warrant further investigations. Although the fetal state of high pulmonary vascular resistance (PVR) permits only 8–10% of cardiac output to reach the lungs (Heymann et al. 1990), postnatal adaptation to air‐breathing life requires a dramatic fall in PVR. The newborn's shift toward pulmonary vasodilation allows a many‐fold increase in blood flow, marking the beginning of the lung's primary role in gas exchange.

### Hyperoxia and cAMP release

Given evidence of crosstalk between cAMP/PKA pathways and PAFR‐mediated signaling (Ibe et al. 2007), we investigated cAMP production by newborn PASMC. Both cAMP and forskolin resulted in a significant cAMP production, indicating the ability of these cells to synthesize cAMP in situ. However, addition of exogenous PAF at physiologic levels did not stimulate the production of cAMP in newborn PASMC, rather production was significantly inhibited. This suggests that in vivo, production of PAF by pulmonary vascular smooth muscle cells, following an appropriate stimulus, will derange the vasodilatory capability of the smooth muscle cells. Platelet‐activating factor and its receptor‐mediated pathways are important to maintain high vasomotor tone in normal fetal pulmonary physiology. We have previously demonstrated that in the fetus PAF levels and PAFR activities are elevated, contributing to this obligatory high PVR (Ibe et al. 1998). We have also shown that immediately following birth, PAF levels fall and PAFR‐mediated effects are decreased, resulting in a decline in PVR (Ibe et al. 1998). When these necessary molecular mechanisms are interrupted or altered, the perinatal pulmonary circulation cannot transition appropriately, manifesting clinically in the newly born infant as hypoxic respiratory failure, specifically PPHN. It has been shown that human infants with PPHN have higher circulating plasma PAF levels and when PPHN resolves, PAF levels decrease (Caplan et al. 1990).

This is the first in vitro study of newborn ovine PASMC where we demonstrate the pathologic effects of both hypoxia and hyperoxia upon PAF receptor‐mediated mechanisms implicated in pulmonary vascular dysfunction. While we hypothesized that with oxygenation at birth, downregulation of PAFR‐mediated signaling is essential for normal newborn pulmonary physiology, we found that both hypoxia and hyperoxia promoted pathologic settings for PAF receptor binding, PAF receptor mRNA and protein expression and PAF receptor‐mediated cell proliferation.

In our previous work on in vitro fetal pulmonary vascular SMC, we not only characterized the consequences of acute and prolonged hypoxia on PAF‐mediated effects but also demonstrated their reversibility with oxygenation (Renteria et al. 2010). In the fetal cells exposed to prolonged state of hypoxia, PAF receptor binding and PAF receptor protein expression were upregulated, in support of the finding that the hypoxic fetal lung environment supports greater PAFR protein expression and therefore greater PAF binding. However, exposure to 30 min of normoxia after a prolonged period of hypoxia immediately downregulated hypoxia‐induced PAFR binding. Given this oxygen tension sensitivity, we suggested that oxygenation would attenuate adverse PAFR‐mediated effects, particularly in newborns whose gas exchange capabilities after birth are compromised.

This study brings caution to the broad assumption of the utility of oxygen in PPHN therapy. For the first time, we have demonstrated that not only exposure to hypoxia but exposure to hyperoxia enhances specific PAFR binding, both in pulmonary artery SMC membranes proteins and in adherent PASMC. In this study, we showed that hyperoxia in newborn membrane proteins augmented PAFR binding almost twice as much as hypoxia. Within adherent newborn PASMC, both acute and chronic hypoxia increased PAFR binding, similar to results in adherent fetal PASMC. In adherent cells, acute exposure to hyperoxia generally did not change binding; however, chronic hyperoxia increased PAFR binding even further than chronic hypoxia.

Previous studies encountered heterogeneity in receptivity of PAFR when comparing fetal with newborn pulmonary artery and vein smooth muscle cells (Renteria et al. 2013b). In this study of newborn PASMC, when treated with specific PAFR antagonist CV3988, PAFR binding was altered by prolonged exposure to different oxygen tensions. While CV3988 inhibited PAFR binding under normoxic conditions, in prolonged hypoxia, CV3988 augmented PAFR binding, suggesting the ability of oxygen tension to induce changes in translational processing of PAFR and its receptivity in perinatal lungs.

In this study, we also investigated the effect of inhibition of endogenous cAMP activity upon PAFR binding. Consistent with counterpart studies in fetal pulmonary arterial and venous smooth muscle cells (Renteria et al. 2013b), we demonstrate that pretreatment with cAMP/PKA antagonist Rp‐cAMPS significantly increased specific PAFR binding in all three oxygen tension conditions over their respective controls. As previously indicated by multiple studies (Stork and Schmitt 2002; Ibe et al. 2007; Renteria et al. 2013b), this is supportive of the importance of the cAMP/PKA axis in counteracting PAF receptor‐mediated signaling.

Given that both hypoxia and hyperoxia increased PAFR binding, we investigated the effects of oxygen tension upon expression of PAFR protein and gene. Our protein expression studies did not demonstrate a straightforward increase in PAFR protein expression when exposed to either hypoxia or hyperoxia that we may have expected. However, prior studies indicate that it is the increased availability of circulating PAF that is the key contributor to PAFR binding (Renteria et al. 2013b). Our studies have shown that fetal lungs synthesize 60% more PAF than newborn lungs and conversely, PAF catabolic activity was 40–60% greater in newborn pulmonary vessels and smooth muscle cells. Furthermore, translational and posttranslational modifications of the PAFR, such as receptor autophosphorylation have been established as important in overall PAFR‐mediated effects, at least in the fetus (Ibe et al. 2002).

Consistent with these findings with western blotting, our qRT‐PCR studies did not demonstrate an increase in *PAFR* gene expression upon exposure to hypoxia or hyperoxia. On the other hand, with addition of physiologic levels of exogenous PAF, *PAFR* gene expression was markedly upregulated by both hypoxia and hyperoxia. This is evidence at the transcriptional level that as an autocoid, PAF upregulates expression of its own receptor and is in line with our previously published studies in fetal ovine PASMC (Ibe et al. 2008).

In fetal pulmonary vascular smooth muscle (PVSMC), we have seen that cyclic nucleotides cAMP and cGMP appear to downregulate PAFR‐mediated effects in PVSMC (Ibe et al. 2007; Hanouni et al. 2015). In turn, hypoxic conditions augment PAFR‐mediated responses and decrease the effects of cyclic nucleotides in the fetus. In this study, we wished to investigate the synergistic effects of cyclic nucleotides and different oxygen tensions on PAF‐mediated signaling in the newborn. As in fetal studies, we also demonstrated in newborn PASMC that cell‐permeable analogs of cAMP and cGMP significantly attenuated PAFR protein expression under normoxic conditions, reinforcing our previously reported proposal of translational regulation of PAFR protein expression (Ibe et al. 2005; Hanouni et al. 2015). However, the converse effect was seen in newborn PASMC exposed to hypoxia, an increased expression of PAFR protein in cells pulsed with both cyclic nucleotide analogs. In other words, this pathologic oxygen tension provides a condition by which cyclic nucleotides fail to downregulate expression of PAFR protein. Our studies in fetus have implicated PAF as a key mediator in creating this pathophysiology. Future studies in newborn PASMC comparing PAF levels produced under different oxygen tension conditions may further elucidate these translational regulatory mechanisms.

We have also previously demonstrated in fetal PVSMC that physiologic levels of exogenous PAF downregulates the expression of both PKG and PKA protein. In this study, we explored the synergistic effects of different oxygen tensions and cyclic nucleotides on PKA‐C*α* protein expression. In normoxia, PKA‐C*α* protein expression was the same in untreated cells and cells treated with cAMP analog. However, treatment with cGMP analog in normoxia augmented PKA‐C*α* protein expression, demonstrating cooperative crosstalk between cAMP‐ and cGMP‐stimulated pathways that has been reported in model cells of intestinal epithelia (Taylor et al. 1998), in porcine coronary microvessels (Kudej et al. 2000), and in rat aortic endothelium (Ray and Marshall 2006). Hypoxia completely obliterated the expression of PKA‐C*α* protein. Again, demonstrating cyclic nucleotide cooperative crossover effect, only treatment with cGMP analog was able to completely rescue expression of PKA‐C*α* protein, while treatment with cAMP analog did not. Hyperoxia increased PKA‐C*α* protein expression over normoxia with no difference observed following treatment with cAMP analog. Of note, in hyperoxia, treatment with cGMP analog resulted in the converse effect than it had under conditions of normoxia and hypoxia – it decreased PKA‐C*α* expression rather than increasing it, indicating oxygen tension‐sensitive translational regulation of PKA‐C*α* protein expression.

We also found that *PKA‐Cα* gene expression decreased significantly following exposure to hypoxia and hyperoxia, to nearly nondetectable levels. Stimulation with PAF also significantly decreased *PKA‐Cα* gene expression in normoxia. However, hypoxia in conjunction with PAF increased *PKA‐Cα* gene expression, suggesting an intersection in PAF/PAFR and PKA/cAMP axes, perhaps a mechanism by which PKA/cAMP is upregulated in an attempt to counterbalance disruption of vascular homeostasis.

The downstream effect of PAF in PASMC proliferation has been well described in PAFR‐mediated signaling in the fetus (Ibe et al. 2008). Here, we demonstrate for the first time the effects of both hypoxia and hyperoxia on proliferation in newborn PASMC. Just as both conditions increased PAFR binding, we show that both hypoxia and hyperoxia increased newborn PASMC proliferation, one mechanism implicated in the pathological vascular remodeling in the fetus (Bixby et al. 2007). In the normal newborn, contractility is important for the transition from intrauterine to air‐breathing postnatal life, but in addition, in newborns affected by PPHN, proliferation does play a critical role. In autopsies of babies who die from PPHN, the pulmonary vasculature clearly demonstrate exaggerated cell growth. A reviewer commented that cell proliferation after birth is less important in PPHN than contractility. We respectfully disagree with this comment. Vessel hypertrophy and hyperplasia play a critic role in the onset and sustenance of PPHN. It has been shown that PPHN is associated with abnormal cell growth in newborn infant with PPHN (Naeye 1961; Naeye and Letts 1962; Haworth and Reid 1976; Murphy et al. 1981). In the worst cases, plexiform lesions are formed (Murphy et al. 1981). Thus, cell growth is a major indicator of PPHN pathology and contributes clinically to PPHN (Naeye 1961; Naeye and Letts 1962; Murphy et al. 1981).

Interestingly, in this study using pharmacologic manipulations with specific PAFR antagonist, we reinforced the previous theory that proliferation in fetal cells is PAFR‐mediated while proliferation in newborn cells is not (Renteria et al. 2013b), but may be secondary to downregulation of cAMP/PKA signaling. However, this was only the case in normoxic conditions. We had previously proposed that there was an inherent difference in the newborn translational or posttranslational processing of PAFR, in comparison with the fetus. In this study, under conditions of hypoxia and hyperoxia, newborn PASMC proliferation appeared to be mediated specifically by endogenous PAFR, in the same manner as shown in fetal cells regardless of oxygen tension. Therefore, it would seem that exposure to either hypoxia or hyperoxia can induce a switch by which newborn PASMC revert back to the fetal state of cell proliferation that is dependent on PAF receptor. This is reminiscent of the old moniker “persistent fetal circulation,” previously used before the term “PPHN.” This becomes problematic because a state that is physiologic in the fetus is conversely pathologic in the air‐breathing newborn.

Seemingly, contradictory effects were also demonstrated in hyperoxia when endogenous cAMP/PKA effects were blocked. In normoxia, treatment with PKA antagonist increased proliferation over baseline, indicating a role of endogenous cAMP/PKA pathway in homeostatic cell growth and preventing hyperplasia. In hyperoxia, a reverse effect was seen, indicating that abnormal oxygen tension adversely affects other signaling pathways, even those mechanisms that would otherwise be protective in normal conditions. Our future studies are designed to further explain this paradoxical relationship, but suffice it to say that hyperoxia causes a disruption in the crosstalk that would be expected between the PAFR‐mediated and cAMP/PKA‐mediated axes.

Essential to perinatal adaptation is the dramatic transition in cellular machinery regulating pulmonary hemodynamics. We speculate that oxygen in the newborn at either sub‐ or supraphysiologic levels creates a setting for PAFR‐mediated vascular dysfunction. Both hypoxia and hyperoxia are pathologic states that disrupt the homeostatic balance of vascular effectors and promote aberrant cell proliferation in the newborn lung.

At every cellular level of PAF receptor signaling, from membrane into cytosol into nucleus, both hypoxic and hyperoxic conditions produce perturbations in PAFR‐mediated responses in newborn ovine PASMC. In addition, abnormal oxygen conditions disrupt the crosstalk with cAMP‐PKA signaling that would otherwise aid in vascular homeostatic balance.

Here, for the first time in newborn pulmonary arterial smooth muscle cells, we confirm the critical importance of maintaining normoxic conditions for the postnatal lung – avoiding both extremes of hypoxia and hyperoxia – to minimize PAF receptor‐driven cellular pathophysiology. Consistent with a growing body of scientific evidence, hyperoxia may be just as harmful as hypoxia in the perpetuation of pulmonary vascular dysfunction and pathogenesis of PPHN.

## Conflict of Interest

None declared.
